# Extractive Fermentation of Lactic Acid in Lactic Acid Bacteria Cultivation: A Review

**DOI:** 10.3389/fmicb.2017.02285

**Published:** 2017-11-20

**Authors:** Majdiah Othman, Arbakariya B. Ariff, Leonardo Rios-Solis, Murni Halim

**Affiliations:** ^1^Department of Bioprocess Technology, Faculty of Biotechnology and Biomolecular Sciences, Universiti Putra Malaysia, Seri Kembangan, Malaysia; ^2^Bioprocessing and Biomanufacturing Research Center, Faculty of Biotechnology and Biomolecular Sciences, Universiti Putra Malaysia, Seri Kembangan, Malaysia; ^3^School of Engineering, Institute for Bioengineering, University of Edinburgh, Edinburgh, United Kingdom

**Keywords:** extractive fermentation, lactic acid bacteria, probiotic, lactic acid, product inhibition, *in situ* removal

## Abstract

Lactic acid bacteria are industrially important microorganisms recognized for their fermentative ability mostly in their probiotic benefits as well as lactic acid production for various applications. Nevertheless, lactic acid fermentation often suffers end-product inhibition which decreases the cell growth rate. The inhibition of lactic acid is due to the solubility of the undissociated lactic acid within the cytoplasmic membrane and insolubility of dissociated lactate, which causes acidification of cytoplasm and failure of proton motive forces. This phenomenon influences the transmembrane pH gradient and decreases the amount of energy available for cell growth. In general, the restriction imposed by lactic acid on its fermentation can be avoided by extractive fermentation techniques, which can also be exploited for product recovery.

## Introduction

For decades, Lactic acid bacteria (LAB) fermentation is found to be applied in dairy industry, wine and cider production, fermented vegetable products production and meat industry (Taskila and Ojamo, 2013). Nowadays, people are aware that diet has an important role in promoting health and preventing disease as a way of spending a healthy lifestyle (Soomro et al., 2002; Pessione, 2012; Nuraida, 2015). Therefore, trend for foods containing probiotic cultures are increasing (Sreekumar et al., 2010). High cell density in cultivations of LAB is crucial in order to get their valuable biomass to be profitably applied as a probiotic ingredient in various products (Schiraldi et al., 2003). Probiotic food products are recommended by the international dairy federation to contain at least 10^6^ to 10^7^ CFU/mL of probiotics at the time of consumption to guarantee its beneficial effects (Halim et al., 2017). Nevertheless, the major problem in the application of LAB culture as probiotics is the reduced growth and biomass concentration owing to end product inhibition (Luedeking and Piret, 2000; Aguirre-Ezkauriatza et al., 2010).

The fermentation of LAB through carbohydrate metabolization produces lactic acid as the major metabolic end-product (Abdel-Rahman et al., 2013). Lactic acid accumulation inhibits LAB growth due to pH alteration into acidic condition. The acidification of cytoplasm and failure of proton motive forces are the reasons for the end product inhibition in LAB fermentation (Wee et al., 2006). As the concentration of lactate increases or the pH of the medium decreases, the concentration of undissociated lactic acid in the medium also increases (Broadbent et al., 2010). The undissociated lactic acid is cytoplasmic membrane soluble and thus can pass through the bacterial membrane via simple diffusion and dissociates inside the cell, whilst the dissociated lactate is insoluble. Eventually, this will affect the transmembrane pH gradient where the transmembrane pH gradient can no longer be maintained and disabled the cellular functions. In addition, the amount of energy that may be used for cell growth also reduces as it is being used for maintaining the transmembrane pH gradient (Wee et al., 2006).

The development of fermentation strategies that can maintain lactate concentration in the culture at below toxic level will be beneficial to overcome the product inhibition (Schiraldi et al., 2003). There are numerous reports on fed-batch fermentation that were conducted to overcome the end product inhibition in LAB fermentation which in turn enhanced biomass production (Boon et al., 2007; Aguirre-Ezkauriatza et al., 2010; Ming et al., 2016). However, the use of fed-batch and pH controlled fermentations for overcoming end product inhibition in LAB fermentations are often inefficient due to high osmotic pressure and the presence of acid anions (Cui et al., 2016). Therefore, to reduce the inhibitory effect of lactic acid during fermentation process, lactic acid must be removed selectively *in situ* from the culture.

## Lab Fermentation Subjected to Product and By-Product Inhibition

The presence of inhibitors known as substrate and product inhibitions that inhibit the cell growth and reduce the product formation activity is one of the main problems in fermentation process (Hujanen et al., 2001; Yuwono et al., 2008; Serrazanetti et al., 2013). Product inhibition in LAB culture is frequently the key reason behind the limited production of biomass observed in batch fermentation. In general, the inhibition by lactic acid can either be competitive or non-competitive inhibition. The effect of lactic acid inhibitory on the cell growth was shown to be stronger than the effect on fermentation activity (Milcent and Carrere, 2001; Madzingaidzo et al., 2002; Zacharof and Lovitt, 2013). The inhibitory effect of lactic acid on cell metabolism and proliferation might be due to the increment in medium osmotic pressure and also other fermentation by-products for example acetic acid, formic acid, or sodium formate that causes an individual inhibitory effect (Lin et al., 2008). Cui et al. (2016) reported that the growth of *Lactobacillus plantarum* in a fed-batch culture was completely inhibited when the osmotic pressure reached 2416 mOsm kg^-1^ due to the continual accumulation of various metabolites and feed medium. It has been reported that there was an inhibition on bacterial growth by lactic acid when the lactic acid was rapidly being produced after the exponential phase of the growth (Monteagudo et al., 1997).

The conventional approach used to overcome product inhibition is by the addition of a base for example calcium hydroxide to neutralize the acid formed and precipitate the insoluble calcium salts (Patel et al., 2008). The insoluble calcium salts will be filtered and treated with sulfuric acid to precipitate calcium sulfate and regenerate the acid. This process, however, consumes high amounts of sulfuric acid and lime and also produces high amounts of liquid and solid wastes that require a costly treatment before being dispose off to the environment. Hetenyi et al. (2011) reported on the use of different compounds which were ammonium hydroxide, sodium hydroxide, calcium carbonate, trimethylamine, and dimethylamine to control the pH of *Lactobacillus* sp. MKT-878 (NCAIM B02375) culture for lactic acid production. Among these tested compounds, trimethylamine was shown to be the best neutralizing agent with the highest lactic acid productivity of 3.13 g L^-1^ h^-1^. Nevertheless, from the technological aspect, it was advisable to use ammonium hydroxide instead.

Another conventional approach for increasing the biomass yield is through the application of fed-batch fermentation. In general, fed-batch fermentation processes can be classified according to the feeding mode such as constant feeding, exponentially feeding, intermittent addition and optimized feeding with or without feedback control (Öztürk et al., 2016; Mears et al., 2017). The process of keeping nutrient concentration below inhibition level by adjusting the feeding rate through fed-batch fermentation may overcome the problem of product inhibition in LAB batch fermentation (**Table 1**). Fed-batch fermentation showed superior performance, in terms of higher biomass and viable cell counts in the freeze-dried product and also lower residual substrate concentrations (Aguirre-Ezkauriatza et al., 2010). The inhibitory effects of glucose on L-lactic acid production by *Lactobacillus lactis* has been avoided and the efficiency of the process has greatly been enhanced when a low level of initial glucose was used and continuously been added during fermentation (Bai et al., 2003). Lee et al. (2007) also demonstrated the feasibility of fed-batch fermentation in overcoming substrate limitation and inhibition and product inhibition while improving the yield of biomass from LAB. Besides, in fed-batch fermentation, the extended lag phase characteristic of low cell density in batch fermentation can be reduced and hence time saving.

**Table 1:** Comparative performances between batch and fed-batch cultures of LAB fermentation.

Microorganisms	Substrates	Biomass production of batch culture		Biomass production of fed-batch culture		Reference
		Biomass production (g/L)	Lactic acid production (g/L)	Biomass production (g/L)	Lactic acid production (g/L)	
*Sporolactobacillus nakayamae*	Sucrose	9	105	14	128	Beitel et al., 2017
*Bacillus coagulans*	Glucose	18	–	25	–	Pandey and Vakil, 2016
*Lactobacillus rhamnosus* B103	Lactose and corn steep liquor	4.79	57	5.5	106	Bernardo et al., 2016
*Lactobacillus salivarius* I 24	Glucose	2.35	29.50	7.114	58.18	Ming et al., 2016
*Lactobacillus rhamnosus* ATCC 10863	Molasses	3	16.5	5.2	22.0	Senedese et al., 2015
*Lactobacillus plantarum* LP02	Glucose	2.53	–	10.12	–	Hwang et al., 2011
*Lactococcus lactis* WICC B-25	Glucose	5.64	4.34	21.34	24.1	Elmarzugi et al., 2010
*Lactobacillus lactis*	Glucose	1.6	200	2.7	210	Bai et al., 2003

## Extractive Fermentation Approaches to Overcome End-Product Inhibition

### Solvent Extraction

Solvent extraction is one of the methods that are commonly used for lactic acid removal (Chen et al., 2012). In solvent extraction process, lactic acid will be first extracted from the culture broth by an extractant followed by lactic acid recovery from the solvent using back extraction into another solvent (Wasewar, 2005). For example, lactic acid extraction method has been developed to simultaneously extract lactic acid using a two-zone fermentor–extractor system (Iyer and Lee, 1999). The method was productively performed under a fed-batch fermentation mode with *in situ* lactic acid removal using a solvent extraction. In general, solvent extraction methods are in fact can be quite difficult as it is not easy to extract lactic acid using common organic solvents due to its hydrophilic nature (Gao et al., 2010). Although alternative method such as reactive extraction has been proposed, the method is however needs high amount of solvents and the toxic effect by the extractants and diluents limits its application. For example, Gao et al. (2009) examined the feasibility of using tri-*n*-decylamine as an extractant in the extractive fermentation of lactic acid by *Saccharomyces cerevisiae* OC-2T T165R. They discovered that high concentration of 1-decylaldehyde in tri-*n*-decylamine was toxic and caused an inhibition effect on the growth of *S. cerevisiae*. Nonetheless the productivity of lactic acid was significantly improved when 1-decylaldehyde in tri-*n*-decylamine was reduced from 700 to 33 ppm.

### Electrodialysis

Electrodialysis fermentation with an ion exchange membrane is frequently used for *in situ* removal of lactic acid, where under the driving force of electrical fields, ions from an aqueous solution will be removed (Habova et al., 2004; Wasewar, 2005). The main applications of this method are to concentrate ionic substances and to remove salts from solutions. Electrodialysis was found to be able to control culture broth in a short time and effectively remove the salt or waste generated from the processes (Datta et al., 1995). Habova et al. (2001) reported on the application of two-stage electrodialysis for *in situ* lactic acid recovery from *Lactobacillus plantarum* L10 fermentation. Lactate was concentrated to 2.5 times (equivalents to 111 g/L) its initial concentration with desalting electrodialysis using ion exchange membranes during the first stage. A final concentration of 157 g/L lactic acid was achieved during the second stage of electroconversion of sodium lactate to lactic acid by water-splitting electrodialysis with bipolar membrane. In the meantime, Kim and Moon (2001) investigated on the direct recovery of lactic acid from fermentation broth using one-stage electrodialysis with three-compartment water-splitting electrodialysis. The system was reported to convert sodium lactate available in the fermentation medium into 96% of lactic acid and 93% of sodium hydroxide. A continuous electrodialysis fermentation system for lactic acid production has been developed and according to this study, the electrodialysis fermentation system with a level meter was the most effective system (Min-Tian et al., 2005). Even though electrodialysis may increase the rate of fermentation, however this method has a few disadvantages such as membrane fouling, high operating cost and deionization of the culture broth (Datta et al., 1995).

### Aqueous Two-Phase Systems

Aqueous two-phase system (ATPS) has received increasing attention in several areas of biotechnology for recovery and purification. In general, the mechanism of ATPS is based on partitioning of biomolecules between two liquid phases commonly formed by mixing a polymer and a salt or two polymers and water (Asenjo and Andrews, 2011; Iqbal et al., 2016). Recently, ATPS has also been intensely applied for lactic acid removal. ATPS consists of a polyelectrolyte, poly(ethyleneimine) (PEI), and a neutral polymer, hydroxyethylcellulose (HEC), was shown to be suitable for lactic acid extractive fermentation (Dissing and Mattiesson, 1994). According to the study, PEI has a positive charge and hence an ion pair can be formed between PEI while lactate was being produced during the fermentation. The lactate produced will be accumulated into the PEI-rich bottom phase as it is formed whereas cells will be accumulated into the HEC top phase or at the interface. Planas et al. (1996) investigated on the long term effect of an ATPS using ethylene oxide-propylene oxide/hydroxypropyl starch polymer-100 on lactate production by overcoming end-product inhibition in a repeated extractive fermentation of *L. lactis* subsp. *lactis* 19435. From the initial 27.8 mM lactate produced in the first batch, the concentrations were increasing with each batch of fermentation until a maximum of 48.1 mM lactate produced in the fifth batch. The final yield recorded was much higher than both the single batch ATPS (35.9 mM lactate) and normal growth medium batch fermentation (38 mM lactate). Later, the use of alcohol/salt ATPS for lactic acid removal was reported by Aydogan et al. (2011). The lactic acid extraction was optimized using a response surface methodology in order to determine the potential of using ethanol/dipotassium hydrogen phosphate for lactic acid recovery. The partition coefficient and extraction yield of lactic acid was found to be up to 2.06 and 80%, respectively. Despite the suitability of the ATPS method for extractive fermentation of LAB, nonetheless the effectiveness of this method is currently limited by the even lactic acid distribution between two phases (Wasewar, 2005) and the high cost of polymers (Aydogan et al., 2011) make it economically unattractive.

### Adsorption

Extractive fermentation using adsorbent can also be conducted to improve LAB fermentation subjected to product and by-product inhibition (**Table 2**). In general, the phenomena of adsorption is described as an accumulation of a gas or liquid solute on the surface of a solid or liquid which form a molecular or atomic film (Okeola and Odebunmi, 2010). Sorption isotherms describe the equilibrium relationship between adsorbent and adsorbate which provide the capacity of an adsorbent for an adsorbate (Ho, 2006). Activated carbon, molecular sieves, polymeric adsorbents and a few other low-cost materials are the examples of common adsorbents used for adsorption techniques (Qiu et al., 2009). Gao et al. (2011) study an extractive fermentation of lactic acid using activated carbon as an adsorbent. The use of activated carbon in this pH-uncontrolled fermentation successfully diminished the inhibitory effect of lactic acid whilst enhanced both productivity and yield. Up to 37 g/kg of lactic acid was recovered from fermentation broth using silicate (zeolite molecular sieves) as an adsorbent (Aljundi et al., 2005). In addition, the yield from this system was maintained with repetitive use. Recently, an extractive fermentation of lactic acid by *Bacillus* strains using Amberlite^TM^ IRA-67 ion exchange resin has been demonstrated under fed-batch fermentation mode (Garret et al., 2015). According to the study, lactic acid productivity for the fed-batch extractive fermentation was found to be 1.31-fold higher than the fed-batch culture without an extractive fermentation system. This observation might be due to the fermentation was occurring below the level of product inhibition. The application of ion exchange resins with bioreactor system offer benefits of overcoming the inhibitory effect of lactate as well as lowering the costs of lactic acid recovery and purification (Monteagudo and Aldavero, 1999).

**Table 2:** *In situ* removal of lactic acid using various solid adsorbents in LAB fermentation.

Adsorbents	Microorganisms	Lactic acid adsorption on adsorbent	Lactic acid production	Biomass production	Reference
WA 30 ion exchange resin	*Streptococcus bovis*	100 mg/g adsorbent	–	–	Yuwono et al., 2017
Amberlite IRA-67	*Lactococcus lactis* ATCC 11454	–	5.9-fold increase in productivity compared with standard batch fermentation without resin)	–	Boonmee et al., 2016
Anion exchange D319	*Lactobacillus plantarum*	–	–	35 g/L (2.3-fold enhancement compared to fermentation without resin)	Cui et al., 2016
Hydrotalcite -type anionic clay	*Streptococcus thermophilus* and *Lactobacillus bulgaricus*	–	3.98 g/L	1.58 × 10^7^ CFU/g	Jinescu et al., 2014
Amberlite IRA-400	*Lactobacillus casei*	–	37.4 g/L	–	Ataei and Vasheghani-Farahani, 2008
Zeolite molecular sieves	*Lactobacillus rhamnosus*	37 g/kg adsorbent	–	–	Aljundi et al., 2005

The *in situ* removal of lactic acid is an innovative process as described by carrying out the fermentation of *Lactobacillus delbrueckii* in a continuous stirred tank fermentor (CSTF) with an ion exchange resins (Monteagudo and Aldavero, 1999). In this method, lactic acid will be adsorbed on solid adsorbents or lactate ion will be adsorbed on ion exchange resins (Wasewar, 2005). By using this method, the maintaining of an actively growing culture in a culture medium of low lactate concentration is made possible. Jianlong et al. (1994) reported on the utilization of weak base D301 anion-exchange resin to reduce lactic acid product inhibition in the extractive fermentation of *Lactobacillus casei*. The lactic acid productivity was found to be improved by 1.47-fold. A method for the removal and recovery of lactic acid from culture broth (i.e., *Lactobacillus delbrueckii, L. bulgaricus*, or *L. leichnanii*) by using anion polymeric adsorbents was patented by Kulprathipanja and Oroshar (1991). They used strong, moderate, and weak basic anion exchange resins to adsorb lactic acid below its pKa. The lone electron pair of the nitrogen atom allows nitrogen atom to form hydrogen bond by sulfate ion. A strongly basic quaternary ammonium ion exchange resin, for example IRA-400 has positive charge and it is able to form ionic bond with sulfate ion. Anion exchange resin with a sulfate form of quaternary ammonium functional group has a weakly basic property and can be used to adsorb lactic acid via acid–base interaction. Therefore, lactic acid adsorption will not affect the inorganic salt in the culture broth (Wasewar, 2005). Nevertheless different types of anion exchange resins often have different affinity toward nutrients available in the fermentation medium (Tan et al., 2011).

An important factor for the successful application of the lactic acid removal system using resin is the selection of resin (Cui et al., 2016). For instance, in order for IRA 67 resin to be effectively applied as lactic acid adsorbent, the resin must have high capacity and selectivity for lactic acid over water and substrates (Gao et al., 2010). This is due to the capacity of IRA 67 resin in lactic acid recovery is lower in fermentation media compared to in aqueous solution of pure lactic acid (John et al., 2008).

Regenerability allows resin to be reused after regeneration or desorption process according to the manufacturer’s instructions (Gao et al., 2010; Cui et al., 2016). Once the resin is saturated with lactic acid, the lactic acid adsorbed can be removed by caustic elution (Garret et al., 2015). In general, the regeneration of weak base ion exchange resin is easier compared to strong base ion exchange resin due to their simple acid base interaction.

The biocompability of resin with microorganism is another important characteristic for resin to possess in order to be used as lactic acid adsorbent (Gao et al., 2010). Most of anion resins show no toxic characteristic to microorganisms, therefore they can be applied directly in bioreactor (Pradhan et al., 2017). In addition, the affinity of cells toward ion exchange resins could easily be understood due to the known chemical composition of the microorganism’s cell wall which responsible for the necessary charges to the cell surface such as diaminopimelic acid, amino acids, or hexosamine (Rotman, 1960).

## Conclusion

Due to the high benefits of LAB to be used as probiotics, it is therefore necessary to improve the performance of LAB fermentation in term of high final biomass concentration. The opportunities can be explored by researchers to invent more alternative methods for lactic acid removal from the culture which can also be used as a part of the lactic acid purification step in the integrated process of fermentation and separation. The application of extractive fermentation techniques in LAB fermentation is expected to produce high cell concentrations and at the same time high *in situ* recovery of lactic acid within the minimum cost.

## Author Contributions

MO designed and wrote the manuscript. AA and LR-S helped in writing and editing. MH critically reviewed, edited, and finalized the manuscript for submission.

## Conflict of Interest Statement

The authors declare that the research was conducted in the absence of any commercial or financial relationships that could be construed as a potential conflict of interest.

## References

[B1] Abdel-Rahman M. A. Tashiro Y. Sonomoto K. (2013). Recent advances in lactic acid production by microbial fermentation processes. *Biotechnol. Adv.* 31 877–902. 10.1016/j.biotechadv.2013.04.002 23624242

[B2] Aguirre-Ezkauriatza E. J. Aguilar-Yáñez J. M. Ramírez-Medrano A. Alvarez M. M. (2010). Production of probiotic biomass (*Lactobacillus casei*) in goat milk whey: comparison of batch, continuous and fed-batch cultures. *Bioresour. Technol.* 101 2837–2844. 10.1016/j.biortech.2009.10.047 20042330

[B3] Aljundi I. H. Belovich J. M. Talu O. (2005). Adsorption of lactic acid from fermentation broth and aqueous solutions on Zeolite molecular sieves. *Chem. Eng. Sci.* 60 5004–5009. 10.1016/j.ces.2005.04.034

[B4] Asenjo J. A. Andrews B. A. (2011). Aqueous two-phase systems for protein separation: a perspective. *J. Chromatogr. A* 1218 8826–8835. 10.1016/j.chroma.2011.06.051 21752387

[B5] Ataei S. A. Vasheghani-Farahani E. (2008). *In situ* separation of lactic acid from fermentation broth using ion exchange resins. *J. Ind. Microbiol. Biotechnol.* 35 1229–1233. 10.1007/s10295-008-0418-6 18712554

[B6] Aydogan O. Bayraktar E. Mehmetoglu U. (2011). Aqueous two-phase extraction of lactic acid: optimization by response surface methodology. *Sep. Sci. Technol.* 46 1164–1171.

[B7] Bai M. Wei Q. Yan Z. H. Zhao X. M. Li X. G. Xu S. M. (2003). Fed-batch fermentation of *Lactobacillus lactis* for hyper-production of L-lactic acid. *Biotechnol. Lett.* 25 1833–1835. 14677707 10.1023/a:1026276925649

[B8] Beitel S. A. Coelho L. F. Sass D. C. Contier J. (2017). Environmentally friendly production of D (-) lactic acid by *Sporolactobacillus nakayamae*: investigation of fermentation parameters and fed-batch strategies. *Int. J. Microbiol.* 2017:4851612. 10.1155/2017/4851612 29081803 PMC5610840

[B9] Bernardo M. P. Coelho L. F. Sass D. C. Contiero J. (2016). L-(+)-lactic acid production by *Lactobacillus rhamnosus* B103 from dairy industry waste. *Braz. J. Microbiol.* 47 640–646. 10.1016/j.bjm.2015.12.001 27266630 PMC4927665

[B10] Boon B. L. Heng J. T. Eng S. C. (2007). Fed-batch fermentation of lactic acid bacteria to improve biomass production: a theoretical approach. *J. Appl. Sci.* 7 2211–2215. 10.3923/jas.2007.2211.2215

[B11] Boonmee M. Cotano O. Amnuaypanich S. Grisadanurak N. (2016). Improved lactic acid production by *in situ* removal of lactic acid during fermentation and a proposed scheme for its recovery. *Arab. J. Sci. Eng.* 41 2067–2075. 10.1007/s13369-015-1824-5

[B12] Broadbent J. R. Larsen R. L. Deibel V. Steele J. L. (2010). Physiological and transcriptional response of *Lactobacillus casei* ATCC 334 to acid stress. *J. Bacteriol.* 192 2445–2458. 10.1128/JB.01618-09 20207759 PMC2863488

[B13] Chen L. Zeng A. Dong H. Li Q. Niu C. (2012). A novel process for recovery and refining of L-lactic acid from fermentation broth. *Bioresour. Technol.* 112 280–284. 10.1016/j.biortech.2012.02.100 22424924

[B14] Cui S. Zhao J. Zhang H. Chen W. (2016). High-density culture of *Lactobacillus plantarum* coupled with a lactic acid removal system with aniom-exchange resins. *Biochem. Eng. J.* 115 80–84. 10.1016/j.bej.2016.08.005

[B15] Datta R. Tsai S. P. Bonsignore P. Moon S. H. Frank J. R. (1995). Technological and economic potential of poly(lactic acid) and lactic acid derivatives. *FEMS Microbiol. Rev.* 16 221–231. 10.1016/0168-6445(94)00055-4Get

[B16] Dissing V. Mattiesson B. (1994). Cultivation of *Lactococcus lactis* in a polyelectrolyte-neutral polymer aqueous two-phase system. *Biotechnol. Lett.* 16 333–338.

[B17] Elmarzugi N. El Enshasy H. Abd Malek R. Othman Z. Sarmidi M. R. Abdel Aziz R. (2010). “Optimization of cell mass production of the probiotic strain *Lactococcus lactis* in a batch and fed-batch culture in pilot scale levels,” in *Current Research, Technology and Education Topics in Applied Microbiology and Microbial Technology* Vol. 2 ed. Mèndez-Vilas A. (Badajoz: Formatex Research Centre) 873–879.

[B18] Gao M.-T. Shimamura T. Ishida N. Nagamori E. Takahashi H. Umemoto S. (2009). Extractive lactic acid fermentation with tri-n-decylamine as the extractant. *Enzyme Microb. Technol.* 44 350–354.

[B19] Gao M. T. Shimamura T. Ishida N. Takahashi H. (2011). pH-Uncontrolled lactic acid fermentation with activated carbon as an adsorbent. *Enzyme Microb. Technol.* 48 526–530. 10.1016/j.enzmictec.2010.07.015 22113026

[B20] Gao Q. Liu F. Zhang T. Zhang J. Jia S. Yu C. (2010). The role of lactic acid adsorption by ion exchange chromatography. *PLOS ONE* 5:e13948. 10.1371/journal.pone.0013948 21085600 PMC2978715

[B21] Garret B. G. Srivinas K. Ahring B. K. (2015). Performance and stability of AmberliteTM IRA-67 ion exchange resin for product extraction and pH control during homolactic fermentation of corn stover sugars. *Biochem. Eng. J.* 94 1–8. 10.1016/j.bej.2014.11.004

[B22] Habova V. Melzoch K. Rychtera M. (2004). Modern method of lactic acid recovery from fermentation broth. *Czech J. Food Sci.* 22 87–94.

[B23] Habova V. Melzoch K. Rychtera M. Pribyl L. Mejta V. (2001). Application of electrodialysis for lactic acid recovery. *Czech J. Food Sci.* 19 73–80. 10.1007/s10529-008-9771-9 18568401

[B24] Halim M. Mustafa N. A. M. Othman M. Wasoh H. Kapri M. R. Ariff A. B. (2017). Effect of encapsulant and cryoprotectant on the viability of probiotic *Pediococcus acidilactici* ATCC 8042 during freeze-drying and exposure to high acidity, bile salts and heat. *LWT Food Sci. Technol.* 81 210–216.

[B25] Hetenyi K. Nemeth A. Sevella B. (2011). Role of pH-regulation in lactic acid fermentation: second steps in a process improvement. *Chem. Eng. Process.* 50 293–299. 10.1016/j.cep.2011.01.008

[B26] Ho Y.-S. (2006). Isotherms for the sorption of lead onto peat: comparison of linear and non-linear methods. *Pol. J. Environ. Stud.* 15 81–86.

[B27] Hujanen M. Linko S. Linko Y. Y. Leisola M. (2001). Optimisation of media and cultivation conditions for L(+)(S)-lactic acid production by *Lactobacillus casei* NRRL B-441. *Appl. Microbiol. Biotechnol.* 56 126–130. 10.1007/s002530000501 11499919

[B28] Hwang C.-H. Chen J.-N. Huang Y.-T. Mao Z.-Y. (2011). Biomass production of *Lactobacillus plantarum* LP02 isolated from infant feces with potential cholesterol-lowering ability. *Afr. J. Biotechnol.* 10 7010–7020. 10.5897/AJB11.507

[B29] Iqbal M. Tao Y. Xie S. Zhu Y. Chen D. Wang X. (2016). Aqueous two-phase system (ATPS): an overview and advances in its applications. *Biol. Proced. Online* 18 18. 10.1186/s12575-016-0048-8 27807400 PMC5084470

[B30] Iyer P. V. Lee Y. Y. (1999). Simultaneous saccharification and extractive fermentation of lignocellulosic materials into lactic acid in a two-zone fermentor-extractor system. *Appl. Biochem. Biotechnol.* 7 409–419. 15304711 10.1385/abab:78:1-3:409

[B31] Jianlong W. Ping L. Ding Z. (1994). Extractive fermentation of lactic acid by immobilized, *Lactobacillus casei* using ion-exchange resin. *Biotechnol. Tech.* 8 905–908.

[B32] Jinescu C. Arus V. A. Pârvulescu O. C. Nistor I. D. (2014). Modelling of batch lactic acid fermentation in the presence of anionic clay. *Food Technol. Biotechnol.* 52 448–458. 10.17113/ftb.52.04.14.3553 27904318 PMC5079144

[B33] John R. P. Nampoothiri K. M. Pandey A. (2008). L(+)-lactic acid recovery from cassava bagasse based fermented medium using anion exchange resins. *Braz. Arch. Biol. Technol.* 51 1241–1248. 10.1590/S1516-89132008000600020

[B34] Kim Y. H. Moon S.-H. (2001). Lactic acid recovery from fermentation broth using one-stage electrodialysis. *J. Chem. Technol. Biotechnol.* 76 169–178. 10.1002/jctb.368

[B35] Kulprathipanja S. Oroshar A. R. (1991). Separation of lactic acid from fermentation broth with an anionic polymeric absorbent. US Patent 5068418 A.

[B36] Lee B. B. Tham H. J. Chan E. S. (2007). Fed-batch fermentation of lactic acid bacteria to improve biomass production: a theoretical approach. *J. Appl. Sci.* 7 2211–2215. 10.3923/jas.2007.2211.2215

[B37] Lin S. K. C. Du C. Koutinas A. Wang R. Webb C. (2008). Substrate and product inhibition kinetics in succinic acid production by *Actinobacillus succinogenes*. *Biochem. Eng. J.* 41 128–135. 10.1016/j.bej.2008.03.013

[B38] Luedeking R. Piret E. L. (2000). A kinetic study of the lactic acid fermentation batch process at controlled pH. *Biotechnol. Bioeng.* 67 393–400. 10.1002/jbmte.390010406 10699846

[B39] Madzingaidzo L. Danner H. Braun R. (2002). Process development and optimisation of lactic acid purification using electrodialysis. *J. Biotechnol.* 96 223–239. 10.1016/S0168-1656(02)00049-4 12044551

[B40] Mears L. Stocks S. M. Sin G. Gernaey K. V. (2017). A review of control strategies for manipulating the feed rate in fed-batch fermentation processes. *J. Biotechnol.* 10 34–46. 10.1016/j.jbiotec.2017.01.008 28179156

[B41] Milcent S. Carrere H. (2001). Clarification of lactic acid fermentation broths. *Sep. Purif. Technol.* 2 393–401. 10.1016/S1383-5866(00)00124-6

[B42] Ming L. C. Halim M. Rahim R. A. Wan H. Y. Ariff A. (2016). Strategies in fed-batch cultivation on the production performance of *Lactobacillus salivarius* I 24 viable cells. *Food Sci. Biotechnol.* 25 1393–1398. 10.1007/s10068-016-0217-1 PMC6049257 30263421

[B43] Min-Tian H. Koide M. Gotou R. Takanashi H. Hirata M. Hano T. (2005). Development of a continuous fermentation system for production of lactic acid by *Lactobacillus rhamnosus*. *Process Biochem.* 40 1033–1036. 10.1016/j.procbio.2004.02.028

[B44] Monteagudo J. M. Aldavero M. (1999). Production of L-lactic acid by *Lactobacillus delbrueckii* in chemostat culture using an ion exchange resins system. *J. Chem. Technol. Biotechnol.* 74 627–634.

[B45] Monteagudo J. M. Rodriguez L. Rincon J. Fuertes J. (1997). Kinetics of lactic fermentation by *Lactobacillus delbrueckii* grown on beet molasses. *J. Chem. Technol. Biotechnol.* 68 271–276.

[B46] Nuraida L. (2015). A review: health promoting lactic acid bacteria in traditional Indonesian fermented foods. *Food Sci. Hum. Wellness* 4 47–55. 10.1016/j.fshw.2015.06.001

[B47] Okeola F. O. Odebunmi E. O. (2010). Freundlich and langmuir isotherms parameters for adsorption of methylene blue by activated carbon derived from agrowastes. *Adv. Nat. Appl. Sci.* 4 281–288. 10.1016/j.ecoenv.2013.05.015 23849465

[B48] Öztürk S. Çalık P. Özdamar T. H. (2016). Fed-batch biomolecule production by *Bacillus subtilis*: a state of the art review. *Trends Biotechnol.* 34 329–345. 10.1016/j.tibtech.2015.12.008 26775901

[B49] Pandey K. R. Vakil B. V. (2016). Development of bioprocess for high density cultivation yield of the probiotic *Bacillus coagulans* and its spore. *J. Biosci. Biotechnol.* 5 173–181.

[B50] Patel M. Bassi A. S. Zhu J. J. X. Gomaa H. (2008). Investigation of a dual-particle liquid–solid circulating fluidized bed bioreactor for extractive fermentation of lactic acid. *Biotechnol. Prog.* 24 821–831. 10.1002/btpr.6 19194893

[B51] Pessione E. (2012). Lactic acid bacteria contribution to gut microbiota complexity: lights and shadows. *Front. Cell. Infect. Microbiol.* 2:86. 10.3389/fcimb.2012.00086 22919677 PMC3417654

[B52] Planas J. Radstrom P. Tjerneld F. Haln-Hagerdal B. (1996). Enhanced production of lactic acid through the use of a novel aqueous two-phase system as an extractive fermentation system. *Appl. Microbiol. Biotehnol.* 45 737–743. 10.1007/s002530050756

[B53] Pradhan N. Rene E. R. Lens P. N. L. Dipasquale L. D’Ippolito G. Fontana A. (2017). Adsorption behaviour of lactic acid on granular activated carbon and anionic resins: thermodynamics, isotherms and kinetic studies. *Energies* 10:665 10.3390/en10050665

[B54] Qiu H. Lv L. Pan B.-C. Zhang Q.-J. Zhang W.-M. Zhang Q.-X. (2009). Critical review in adsorption kinetic models. *J. Zhejiang Univ. Sci. A* 10 716–724. 10.1631/jzus.A0820524

[B55] Rotman B. (1960). Uses of ion exchange resins in microbiology. *Bacteriol. Rev.* 24 251–260. 14439487 10.1128/br.24.2.251-260.1960 PMC440993

[B56] Schiraldi C. Adduci V. Valli V. Maresca C. Giuliano M. Lamberti M. (2003). High cell density cultivation of probiotics and lactic acid production. *Biotechnol. Bioeng.* 82 213–222. 10.1002/bit.10557 12584763

[B57] Senedese A. L. C. Filho R. M. Maciel M. R. W. (2015). L-Lactic acid production by *Lactobacillus rhamnosus* ATCC 10863. *ScientificWorldJournal* 2015:501029. 10.1155/2015/501029 25922852 PMC4397478

[B58] Serrazanetti D. I. Gottardi D. Montanari C. Gianotti A. (2013). “Chapter 23 Dynamic stress of lactic acid bacteria associated to fermentation processes,” in *Lactic Acid Bacteria — R & D for Food, Health and Livestock Purposes* ed. Kongo M. (Rijeka: Intech Open Science) 10.5772/51049

[B59] Soomro A. H. Masud T. Anwaar K. (2002). Role of lactic acid bacteria (LAB) in food preservation and human health-A review. *Pak. J. Nutr.* 1 20–24. 10.3923/pjn.2002.20.24

[B60] Sreekumar G. Krishnan S. Prathipa R. C. (2010). Studies on the effects of end product inhibition over lactic acid bacteria under high cell density cultivation process. *Int. J. Chem. Sci.* 8 92–99.

[B61] Tan J. S. Ramanan N. R. Ling T. C. Shuhaimi M. Ariff A. B. (2011). Enhanced production of periplasmic interferon alpha-2b by *Escherichia coli* using ion-exchange resin for *in situ* removal of acetate in the culture. *Biochem. Eng. J.* 58–59 124–132. 10.1016/j.bej.2011.08.018

[B62] Taskila S. Ojamo H. (2013). “The current status and future expectations in industrial production of lactic acid by lactic acid bacteria,” in *Lactic Acid Bacteria - R & D for Food, Health and Livestock Purposes* ed. Kongo J. M. (Rijeka: INTECH Open Access Publisher) 615–632.

[B63] Wasewar K. L. (2005). Separation of lactic acid: recent advances. *Chem. Biochem. Eng. Q.* 19 159–172.

[B64] Wee Y. J. Kim J. N. Ryu H. W. (2006). Biotechnological production of lactic acid and its recent applications. *Food Technol. Biotechnol.* 44 163–172.

[B65] Yuwono S. D. Ghofar A. Kokugan T. (2008). Effect of product inhibitions on L-lactic acid fermentation from fresh cassava roots in tofu liquid waste by *Streptococcus bovis*. *Japan J. Food Eng.* 9 59–65.

[B66] Yuwono S. D. Nugroho R. H. Mulyono Buhani Suharso Sukmana I. (2017). Purification of lactic acid from cassava bagasse fermentation using ion exchange. *APRN J. Eng. Appl. Sci.* 12 3853–3857.

[B67] Zacharof M.-P. Lovitt R. W. (2013). Modelling and simulation of cell growth dynamics, substrate consumption, and lactic acid production kinetics of *Lactococcus lactis*. *Biotechnol. Bioprocess Eng.* 18 52–64. 10.1007/s12257-012-0477-4

